# Combinatorial study of Fe-Co-V hard magnetic thin films

**DOI:** 10.1080/14686996.2017.1287520

**Published:** 2017-03-20

**Authors:** Sean W. Fackler, Vasileios Alexandrakis, Dennis König, A. Gilad Kusne, Tieren Gao, Matthew J. Kramer, Drew Stasak, Kenny Lopez, Brad Zayac, Apurva Mehta, Alfred Ludwig, Ichiro Takeuchi

**Affiliations:** ^a^ Department of Materials Science & Engineering, University of Maryland, College Park, MD, USA; ^b^ Materials Measurement Science Division, Institute for Materials, Ruhr-Universität Bochum, Bochum, Germany; ^c^ National Institute of Standards and Technology, Gaithersburg, MD, USA; ^d^ Ames Laboratory and Materials Science and Engineering, Iowa State University, Ames, IA, USA; ^e^ Stanford Synchrotron Radiation Lightsource/SLAC, Stanford University, Menlo Park, CA, USA

**Keywords:** Vicalloy, permanent magnet, high-throughput, combinatorial thin film, 50 Energy Materials, 306 Thin film / Coatings, 203 Magnetics / Spintronics / Superconductors

## Abstract

Thin film libraries of Fe-Co-V were fabricated by combinatorial sputtering to study magnetic and structural properties over wide ranges of composition and thickness by high-throughput methods: synchrotron X-ray diffraction, magnetometry, composition, and thickness were measured across the Fe-Co-V libraries. In-plane magnetic hysteresis loops were shown to have a coercive field of 23.9 kA m^–1^ (300 G) and magnetization of 1000 kA m^–1^. The out-of-plane direction revealed enhanced coercive fields of 207 kA m^–1^ (2.6 kG) which was attributed to the shape anisotropy of column grains observed with electron microscopy. Angular dependence of the switching field showed that the magnetization reversal mechanism is governed by 180° domain wall pinning. In the thickness-dependent combinatorial study, co-sputtered composition spreads had a thickness ranging from 50 to 500 nm and (Fe_70_Co_30_)_100-x_V_x_ compositions of x = 2–80. Comparison of high-throughput magneto-optical Kerr effect and traditional vibrating sample magnetometer measurements show agreement of trends in coercive fields across large composition and thickness regions.

## Graphical abstract

**Figure:**
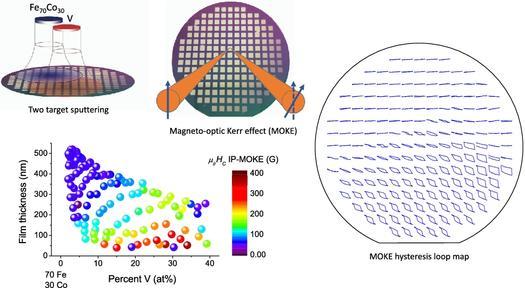

## 1. Introduction

### 1.1. Background

Modern society relies on permanent magnets that are common in motors, generators, speakers, and actuators. High-performance permanent magnets used in efficient and compact devices all contain one or more rare-earth elements [1]. In 2011 rare-earth element prices increased to nearly 10 times their 2010 levels, which prompted research into alternative permanent magnets that do not contain rare-earth elements [2]. Requirements for a permanent magnet include a large magnetization, high Curie temperature, large magnetic anisotropy, and a large coercive field.

We have studied alloys of Fe and Co with refractory metals including W [3], Mo [4], Nb [5], and V (this work) as part of our search for rare-earth-free permanent magnets using the combinatorial methodology. These refractory metal additions are stable at a temperature of 200°C, as required for traction motors. Studies have suggested that refractory additions have strong spin-orbit coupling that may give FeCo a large magnetocrystalline anisotropy through hybridization [6–9].

The Fe-Co-V ternary system in particular is known to contain a permanent magnet called Vicalloy with nominal composition of Fe_40_Co_52_V_8_ [10, 11], The origin of Vicalloy’s coercive field of 23.9 kA m^–1^ (300 G) is qualitatively understood as a dispersion hardened or precipitate hardened magnet that requires the formation of precipitates to obtain the coercive field. However, to our knowledge the precipitate hardening effect is not quantitatively understood in Vicalloys [11–14]. Vicalloy I is defined as alloys with nominal composition Fe_40_Co_52_V_8_ requiring an annealing at 600°C after casting to achieve a maximum energy product of 8 kJ m^–3^ (1 MGOe) [10].

Vicalloys are known to be sensitive to cold-working deformation, which can increase their maximum energy product. A second alloy called Vicalloy II requires considerable cold-working of over 90% and increased V content of 12 to 16 atomic percent (at%) for a maximum energy product about three times larger than Vicalloy I [12].

A shape anisotropy contribution to coercive field for Vicalloy I was ruled out after electron microscopy images showed only circular particles [12]. A uniaxial anisotropy from strain was also ruled out after careful X-ray diffraction measurements did not show residual strain [12]. Magnetocrystalline anisotropy still remains a possibility because of an inconclusive Mössbauer study that could not distinguish s-electron and d-electron contributions to changing Mössbauer spectra [14]. A more recent investigation of Vicalloy containing 7 at% V claimed that the B2 ordering of FeCo was responsible for the coercive field. A coercive field of half the expected value was found in the ordered alloy without precipitates [11]. The same researchers proposed that a pinning site at the B2 anti-phase boundaries (APBs) was responsible for the increase in coercive field observed without providing quantitative support.

### 1.2. Study overview

The present study has two aims. First, we used the combinatorial thin film method to study the structural and magnetic properties of the Fe-Co-V thin film system (Section 2). Second, we compared coercive fields measured by both high-throughput (HiTp) magneto-optical Kerr effect (MOKE) and the slower more traditional vibrating sample magnetometer (VSM) over large composition and thickness ranges (Section 3).

Specifically, for the first aim we provide: (1) the synthesis methods of the combinatorial composition libraries and experimental details; (2) results of HiTp synchrotron X-ray diffraction (XRD) providing the dependence of crystal structure versus composition; (3) transmission electron microscopy (TEM) results showing the microstructure of a representative sample in the library; (4) VSM results giving hysteresis loops and composition dependence of the energy product; and (5) a review of the Kondorsky model along with a comparison to angular dependent switching field measurements revealing the magnetization reversal mechanism.

For our second aim (Section 3), we compared the in-plane coercive field measured by HiTp MOKE and regular VSM measurements across large film thickness and composition ranges of the Fe_70_Co_30_-V sub-system. This thickness dependence study was performed to qualitatively understand the effects of using the combinatorial thin film method. This is important because magnetic films are known to exhibit dramatic changes in properties versus thin film thickness [15, 16], and ultimately the knowledge discovered with the combinatorial thin film method should be applied to processing bulk permanent magnets.

Another motivation for the thickness dependent study is to better understand the utility of MOKE screening compared to more traditional VSM measurements. The reliability of HiTp MOKE as a non-destructive screening technique for studying permanent magnetic thin films needs to be determined since it is a surface sensitive technique compared to VSM’s volume sensitivity. We also believe that the combinatorial method is an underutilized methodology to study magnetism and hence published information showing the efficacy of HiTp magnetometry methods should help build a good reputation for the combinatorial method [17].

We offer the data related to this work as hosted by two materials science databases for further viewing, dissemination, sharing, and analysis: Citrination (citrination.com/fe-co-v) and MPContribs [18]. The dataset was contributed to Materials Project [19] for public dissemination via the contribution framework MPContribs (P. Huck, private communication, 2017) and can be reached at the webpage materialsproject.org/mpcontribs/fe-co-v/dataset-01. The public sharing of materials science data meets federal regulations for the accessibility of publically funded research. We also believe it encourages further collaboration and scientific discovery among scientists inside and outside of materials science.

## 2. Structural and magnetic properties of the Fe-Co-V thin film library

### 2.1. Synthesis and experimental methods

One Fe-Co-V materials library was co-sputtered in Ruhr University Bochum, Germany (RUB) on a 100 mm Si wafer and one was co-sputtered at University of Maryland (UMD) on a 75 mm Si wafer. We designate these materials libraries RUB and UMD1, respectively. The fabrication of materials libraries at different institutions was to check the reproducibility of the combinatorial technique. The details of the composition spread techniques by co-sputtering at RUB [20] and UMD [21] are described elsewhere. A third materials library was co-sputtered at UMD on a 75 mm Si wafer with a large thickness range and designated as UMD2. The composition and thickness spread was achieved by co-sputtering a Fe_70_Co_30_ alloy target and an elemental V target. The geometry of the chamber and different sputtering rates of the targets determine the composition and thickness variation that is known as a natural spread.

The materials libraries were prepared in downward facing DC magnetron co-sputtering systems (base pressure < 3× 10^−6^ Pa) at room temperature on Si wafers. The Si wafers have a 200–500 nm SiO_2_ coating that serves as a diffusion barrier. It is known from silicate phase diagrams that any possible chemical reaction is unlikely at the annealing temperature of 600 to 700°C [22–24]. A sharp film/substrate interface shown in cross-sectional TEM images also shows no deleterious reactions occurred between film and substrate.

The natural spread library of the Fe_70_Co_30_-V alloy sub-system ranged from 50 to 500 nm. The RUB materials library was a continuous thin film while the materials libraries made at UMD used a shadow mask to separate the films into a grid of 4.5 mm × 4.5 mm regions making thickness measurements with a profilometer possible. The room temperature deposited materials libraries were subsequently annealed in vacuum at temperatures between 600 and 700°C. The average composition of each 4.5 × 4.5 mm thin film region was determined by energy-dispersive X-ray spectroscopy (EDS) for library RUB and wavelength-dispersive X-ray spectroscopy (WDS) for the UMD libraries. Quantitative X-ray microanalysis techniques are known to be accurate to ±1 at% [25]. Table 1 shows atomic composition in whole numbers for EDS measurements on materials library RUB. WDS has a lower detection limit than EDS so composition values for libraries UMD1 and UMD2 measured with WDS include the extra significant figure. Table 1 summarizes the composition and thickness ranges for the three material libraries studied.

**Table 1.** Materials libraries compositions and thicknesses.

Composition and thickness range	RUB – Fe-Co-V library (RUB)	UMD – Fe-Co-V library (UMD1)	UMD – Fe_70_Co_30_-V library (UMD2)
Fe range (at%)	17–70	9.5–94.8	13.0–68.5
Co range (at%)	22–65	3.5–87.5	5.2–30.2
V range (at%)	7–29	0.7–42.2	2.0–81.8
Thickness range (nm)	250–400	250–400	50–500

After annealing we used XRD to map the crystal structure of each sample on the materials library at beam line 1–5 of Stanford Synchrotron Radiation Laboratory using an X-ray energy of 16.5 keV corresponding to a wavelength of 0.7514 Å. The HiTp MOKE test stand was used to rapidly map magnetic hysteresis loops across the materials library. The RUB materials library and select regions of the UMD materials libraries were cut into small pieces to measure the magnetic and microstructural properties by VSM and TEM, respectively.

### 2.2. Synchrotron X-ray diffraction

**Figure 1.**
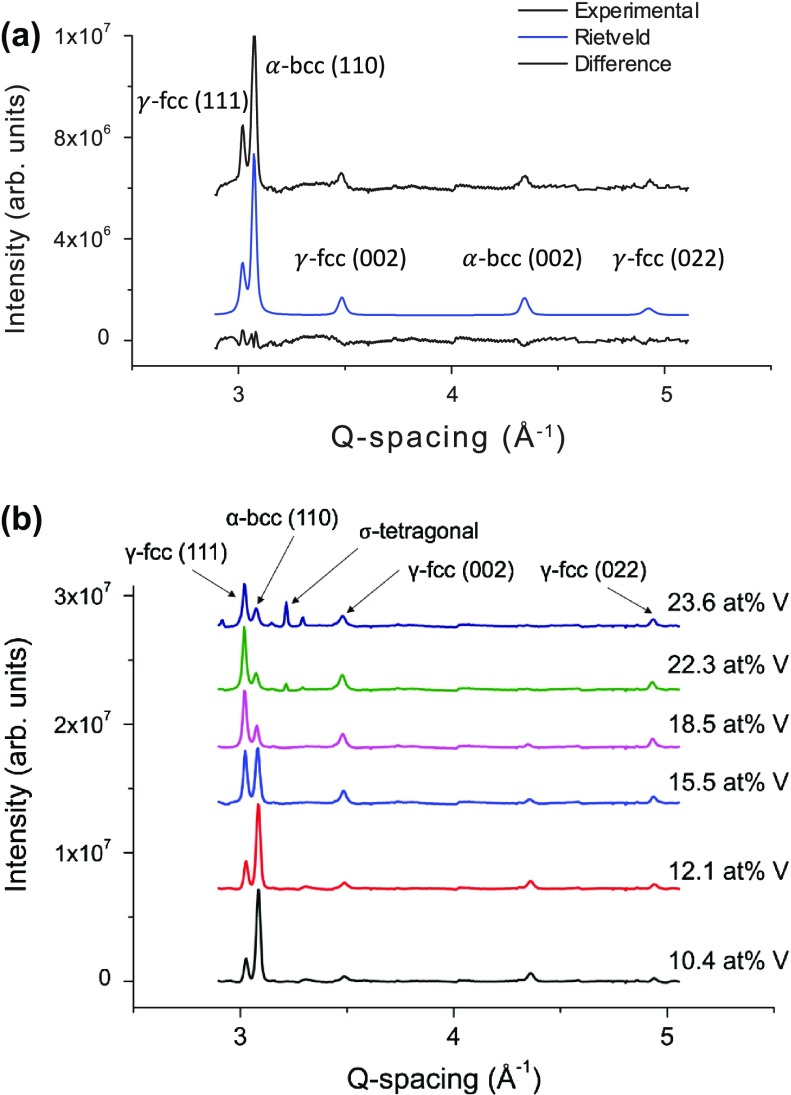
(a) Synchrotron XRD results showing the experimental Rietveld refinement and residuals of an Fe_56_Co_23_V_11_ thin film. Known phases of the α body-centered cubic phase (α-bcc) with a strong (110) preference and the γ face-centered cubic (γ-fcc) phase were identified. (b) XRD patterns of thin Fe-Co-V films with equal atomic concentrations of Fe and Co and varying V content. The clear presence of the σ-tetragonal phase is found above 20 at% V in agreement with bulk phase diagrams.

Synchrotron XRD on the ternary composition spread revealed an agreement of crystal structure and lattice spacing with known bulk samples. Figure 1(a) shows the experimental XRD data and the Rietveld refinement with residuals of a representative Vicalloy sample from the RUB library. The face-centered cubic γ-phase and the body-centered cubic α-phases were included in the Rietveld refinement. Determined lattice spacings of *a*_*γ*_ =  3.615 Å and *a*_*α*_ = 2.900 Å agree with literature [26]. Figure 1(b) shows that in addition to the Vicalloy composition region it was also confirmed that the tetragonal σ-phase appears for compositions containing above 20 at% V with equal atomic concentration of Fe and Co [27]. We did not observe any superlattice Bragg peaks associated with the ordered B2 phase in our synchrotron diffraction patterns despite adequate signal to noise ratio. XRD measurements between libraries RUB and UMD1 (not shown) showed agreement, hence the crystal structure for the Fe-Co-V material libraries are consistent with the known equilibrium phase diagram [26].

**Figure 2.**
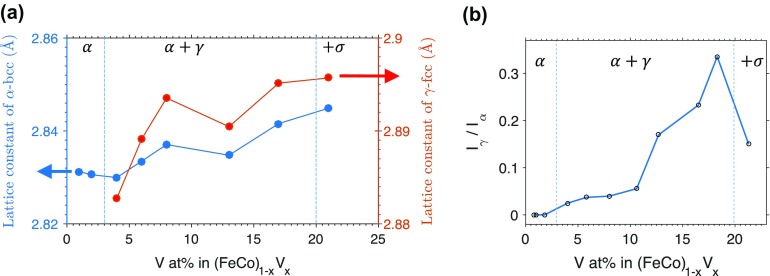
(a) Dependence of lattice parameters on V concentration for α-bcc and γ-fcc phases of Fe-Co-V samples with nominally equal atomic concentrations of Fe and Co. (b) Ratio of the diffraction peak intensities for the γ-fcc (111) peak divided by the α-bcc (110) peak versus V composition. In (a) and (b) vertical dashed lines are boundaries separating various phase regions labeled in Greek letters.

Figure 2(a) shows the evolution of the lattice parameters for α-bcc and γ-fcc phases versus vanadium composition while holding Fe and Co within 5 at% of equal atomic concentrations. We observe an expansion of both unit cells as vanadium content increases. Figure 2(b) shows that the ratio of γ-fcc (111) to α-bcc (110) peak intensity increases as vanadium content increases. It appears both lattice parameters increase anomalously and γ-fcc phase fraction shows a change in slope versus V content near 8 at% V.

### 2.3. TEM characterization

**Figure 3.**
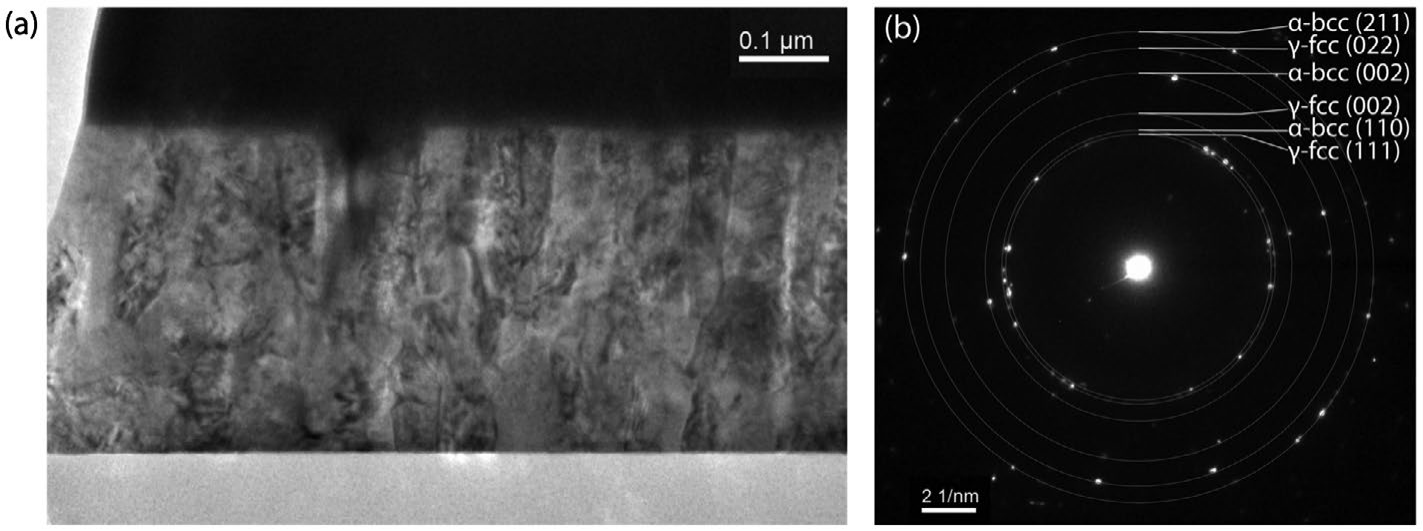
(a) Cross-sectional TEM image indicates columnar grain growth in the Fe_53_Co_38_V_9_ thin film. (b) SAED pattern shows the high crystallinity of the film. Gray rings indicate the various indices of the α-bcc and γ-fcc phases labelled in white.

One sample at the nominal Vicalloy composition of Fe_53_Co_38_V_9_ was studied with TEM to investigate the detailed microstructural properties. Figure 3(a) shows the cross-sectional TEM image of the Vicalloy thin film and Figure 3(b) shows the selected area electron diffraction (SAED) image indicating the high crystallinity of the film. The TEM image reveals what appear to be columnar grains. The dark contrast at the top of Figure 3(a) is a protective tungsten layer from lamella preparation. Due to the small grain size and limitations of the TEM beam size (smallest aperture), the SAED patterns included both γ-phase and α-phases making it impossible to distinguish the arrangement of the individual phases with dark-field TEM. The SAED images were integrated radially and were consistent with synchrotron XRD results above.

### 2.4. Magnetic hysteresis loops and energy product

**Figure 4.**
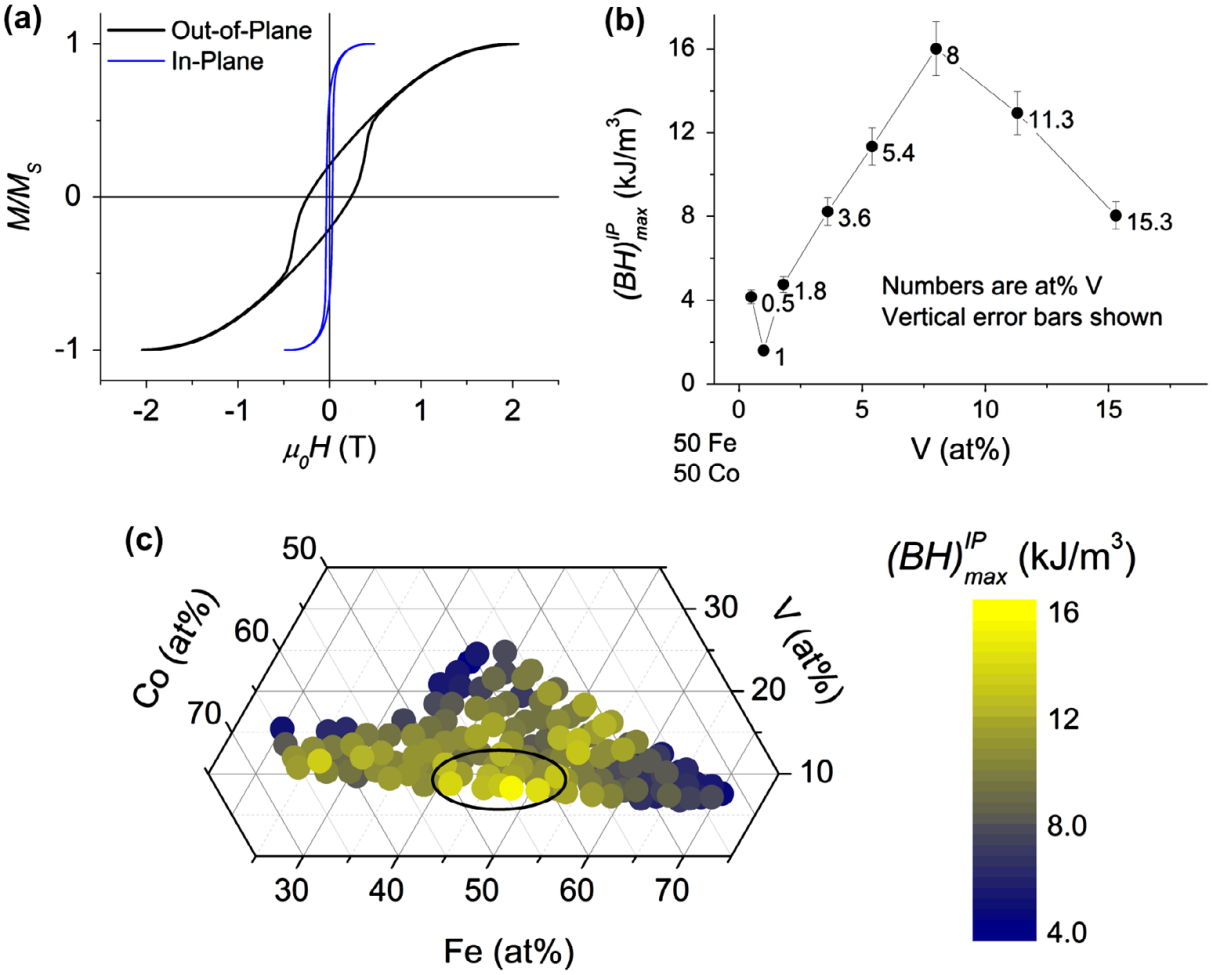
(a) VSM results for in-plane and out-of-plane *M*(*H*) loops of an Fe_38_Co_53_V_9_ sample. Data were normalized to facilitate comparison of saturation for in-plane and out-of-plane loops. (b) In-plane (*BH*)_max_ values were calculated from VSM hysteresis loops from library UMD1 and plotted versus the V content of alloys with equal atomic concentrations of Fe and Co. Each point is labeled with at% V and the error in (*BH*)_max_ is shown as vertical bars. (c) (*BH*)_max_ map values were calculated from in-plane VSM loops of library RUB. The (*BH*)_max_ values were color mapped and plotted on the ternary composition diagram for the Fe-Co-V system. The nominal Vicalloy region is circled in black as studied by Nesbitt [10].

Room temperature magnetic hysteresis loops of the films were measured with VSM by cutting individual samples from the materials libraries. Figure 4(a) shows the representative in-plane and out-of-plane hysteresis loops for a Vicalloy thin film. As seen in Figure 4(a) the out-of-plane coercive field is 207 kA m^–1^ (2.6 kG). Out-of-plane coercive fields of over 79.6 kA m^–1^ (1 kG) were observed over large Fe-Co composition regions below 10 at% V. The in-plane hysteresis loop showed a coercive field equal to 23.9 kA m^–1^ (300 G), and the expected magnetization of nominally 1000 kA m^–1^ [10]. We claim that the increase in out-of-plane coercive field of the Fe-Co-V films is due mainly to shape anisotropy from elongated grains as observed in cross-sectional TEM images in Figure 3(a). The effect of columnar grain shape on out-of-plane magnetic anisotropy has been observed previously in other films such as Ni-Fe [28] and NdFeB [29], but not in Fe-Co-V films before. Further studies will be needed to confirm this finding.

Analogous to the maximum energy product used as a figure of merit for permanent magnets, we calculated the maximum (*BH*) product, (*BH*)_max_, for films with different compositions [30]. Figure 4(b) shows a plot of (*BH*)_max_ versus V composition containing equal atomic concentrations of Fe and Co. A peak is clearly seen at 8 at% V, which agrees with Vicalloy I compositions studied by Nesbitt [10]. Figure 4(c) shows the (*BH*)_max_ map calculated from the in-plane (IP) VSM hysteresis loops versus the composition in the ternary diagram. The map reveals a peak of (*BH*)_max_ at the expected Vicalloy composition region. Values from Figure 4(b) and 4(c) are from libraries UMD1 and RUB1, respectively. Their agreement shows the reproducibility of combinatorial synthesis carried out for two completely different sputtering systems.

### 2.5. Kondorsky model

**Figure 5.**
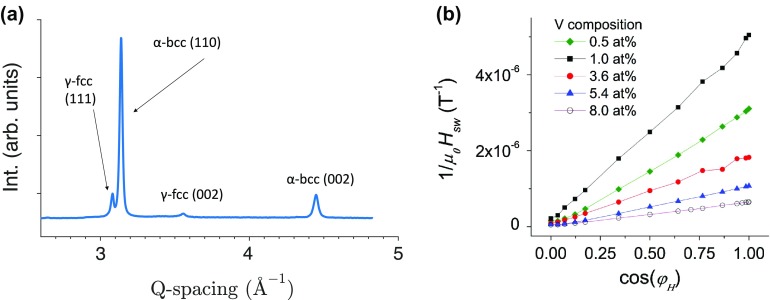
(a) Plot showing the experimental angular dependence of switching field for an Fe_51_Co_41_V_8_ sample in black dots and a fit with the Kondorsky model in red. (b) Angular dependence of the inverse switching field for compositions from 0.5 at% V to 8 at% V with nominally equal concentrations of Fe and Co. Inset of (a) shows the XRD pattern of the same sample as the given angular dependent switching field data.

We now use the Kondorsky model to explain the magnetic switching mechanism found in the samples. The Kondorsky model is defined using the energy required for a 180° domain wall to move past an obstruction, or pinning site [31]. The in-plane film direction is defined as the angle, *φ*_*H*_ = 0°, while the out-of-plane direction is *φ*_*H*_ = 90°. The Kondorsky model says when a field is applied at any direction away from the easy axis, only the component of the external field along the easy axis contributes to the depinning of domain walls, hence a cosine term. The angular dependence is described by the inverse cosine relation of the switching field *μ*_0_ *H*_*sw*_ to the out-of-plane angle *ϕ*_*H*_ such that $\mu_{0}H_{sw}\left(\varphi_{H}\right)=\mu_{0}H_{sw}\left(0^{\circ }\right)/\text{cos}\left(\varphi_{H}\right)$. This relation is common in the description of magnetic media [32] and is shown in Figure 5(a).

Figure 5(b) shows the angular dependence of the inverse switching fields for the same equiatomic Fe-Co alloys with increasing V content from Figure 4(b). The inverse switching field 1/*μ*_0_ *H*_*sw*_ is plotted versus $\cos\left(\varphi_{H}\right)$. A linear relationship of the data shows strong agreement with the Kondorsky model as in Figure 5(a). The good agreement of the experimental data with the Kondorsky model is evidence that the magnetization reversal mechanism is dominated by domain wall motion and pinning. Even at low V content of 2 at%, the γ-fcc precipitate phase is found, which may explain why all the samples in Figure 5(b) agree with the Kondorsky model [33]. Discrepancies between experimental data and the Kondorsky relation at the highest $\varphi_{H}$are attributed to misalignment of the sample angle to within *φ*_*H*_ ± 2°.

The domain wall pinning effect supported by the Kondorsky model is evidence that the precipitate hardening effect is likely from the cited γ-fcc precipitate phase acting as pinning sites for domain wall motion [5, 34] The scientific community largely accepts the idea; however, little hard evidence supports the claim other than correlation of the coercive field with the formation of precipitates. Further studies should include confirmation of the proposed 180° pinning mechanism. Although Zakharov et al. [11] proposed pinning of an ordered B2 phase on the APBs, we propose a pinning mechanism related to grain size and non-magnetic inclusions of the γ-fcc phase as per the models of Bernasconi et al. [35] and Paul [36]. Indeed, it has been recognized that understanding the microstructure, and hence distribution of grains, grain boundary compositions, and secondary phases is important for engineering magnetic materials [37].

## 3. VSM and MOKE comparison

**Figure 6.**
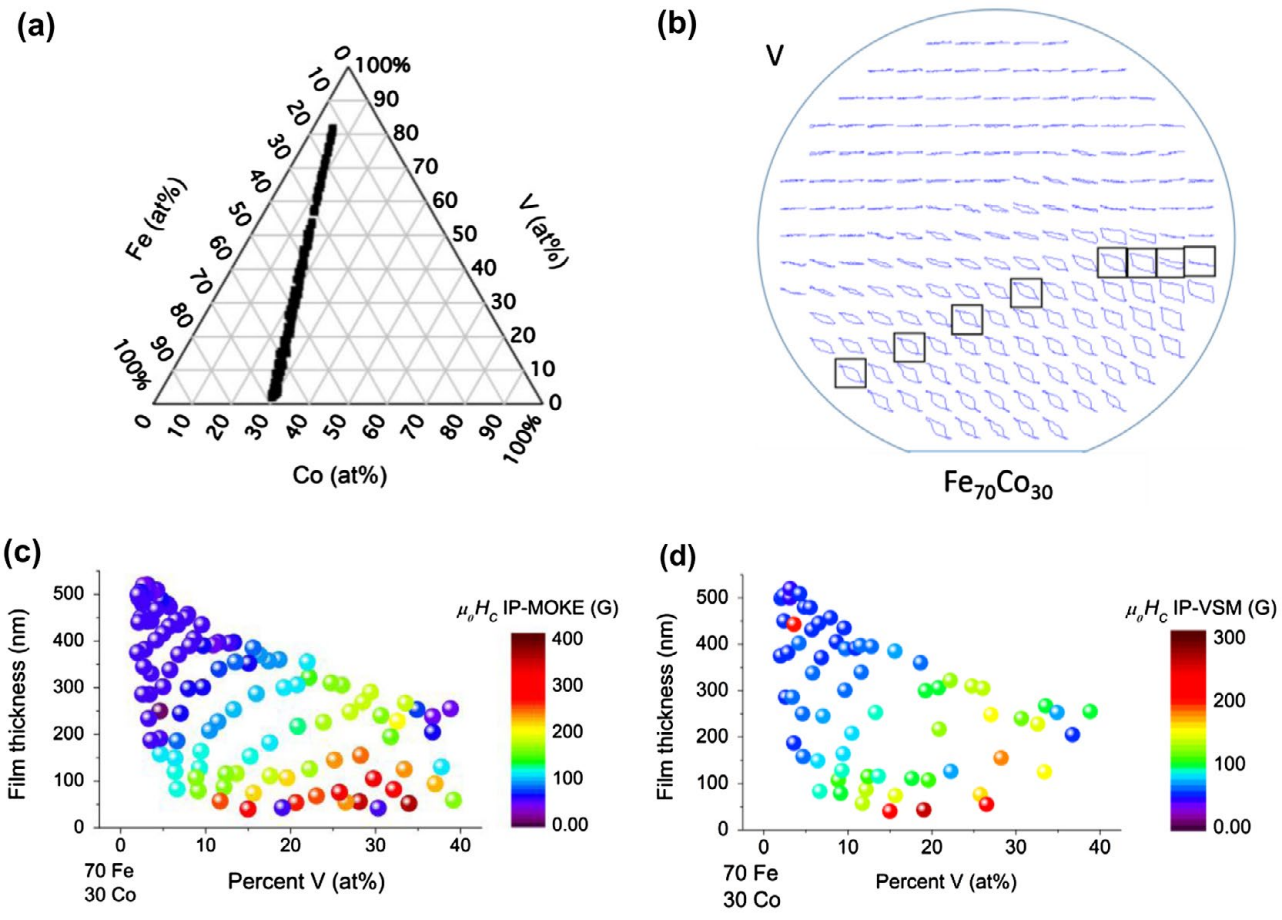
(a) Ternary composition diagram of the Fe_70_Co_30_-V natural thickness gradient from library UMD2. (b) Out-of-plane MOKE hysteresis loop map of the same natural thickness spread. Each MOKE hysteresis loop is positioned at its respective position on the wafer which is depicted with the wafer flat at bottom. The field range of each loop is 400 kA m^–1^ (5 kOe). Hysteresis loops of the nominal composition Fe_65_Co_26_V_9_ are boxed where decreasing film thickness goes from left to right. (c) In-plane MOKE coercive field (*μ*_0_ *H*_*C*_) map of thickness versus V composition where coercive field is color mapped. (d) In-plane VSM coercive field map with thickness versus V composition.

The second aspect of the combinatorial study is to systematically compare VSM and MOKE data across large composition and thickness ranges. Figure 6(a) shows the ternary composition diagram for the natural thickness gradient library UMD2 with a large composition gradient between V and the Fe_70_Co_30_ alloy target. Figure 6(b) shows the out-of-plane MOKE hysteresis loops positioned at their corresponding sample position on the wafer where the field range of each hysteresis loop is 400 kA m^–1^ (5 kOe). Boxes indicate samples of the same nominal composition of Fe_65_Co_26_V_9_ but with decreasing thickness from left to right. High V content samples near the top left of 6(b) are paramagnetic or weak ferromagnets because of the flat MOKE response. The utility of the MOKE hysteresis map is in quickly identifying regions of interest for further study.

Figure 6(c) shows the coercive fields from each of the in-plane MOKE loops color-coded on a thickness vs. composition diagram. Figure 6(d) shows in-plane VSM hysteresis loops for selected samples to directly compare MOKE and VSM results. Despite the slight difference in scales for the coercive fields there is a good agreement of the trend for coercive fields over large composition and thickness ranges. The relative coercive field of the thicker samples with low V contents agrees well between the two plots. For thinner films with higher V content the trend of large coercive fields (red points) is also preserved between the plots. For films with V > 20 at%, the coercive field suddenly rises and then gradually decreases until samples with V contents > 35 at% exhibit coercive fields below 4 kA m^–1^ (50 G). We showed that trends of coercive field for in-plane measurements along the easy axis of the film agree well between VSM and MOKE measurements, allowing quick screening for regions of interest for further study with the slower VSM technique.

## 4. Discussion of future work

Zakharov et al. [11] postulated pinning of an ordered B2 phase on the anti-phase boundaries to explain their observed maximum coercive field of 9.2 kA m^–1^ (116 Oe) for Vicalloy having 7 at% V. The role of the order in FeCo alloys has been important in optimizing soft magnetic and mechanical properties [38] and may also contribute to the high coercive field of Vicalloy [39].

To explain the measured coercive field at V concentrations of 8–10 at%, 23.9 kA m^–1^ (300 G), we find it necessary to consider effects beyond APB pinning. We propose a pinning mechanism related to grain size and precipitates of the γ-phase. Paul [36] developed a simple domain wall pinning model where the size of the magnetic defect and the ratio of the magnetic properties like magnetization, anisotropy constant, and exchange energy between the two phases are considered. The analytical method can be used for initial analysis and then micromagnetic simulations can consider the more complicated effects of three dimensional stray fields, misaligned grains, and reduced anisotropies [40]. Finally, with a more detailed dark-field TEM study to learn about the precise phase distribution, one could apply micromagnetic simulations to engineer the microstructure of this precipitate hardened permanent magnet Vicalloy.

## 5. Conclusions

We performed combinatorial mapping of composition and thickness gradient Fe-Co-V thin film libraries using high-throughput synchrotron X-ray diffraction, MOKE, and WDS to understand structure, property, and composition relationships in these hard magnetic materials, respectively. We showed the in-plane coercive field of 23.9 kA m^–1^ (300 G) and 1000 kA m^–1^ magnetization for the known Vicalloy composition of 8 at% V with nominally equal atomic concentrations of Fe and Co. Reproducibility of the combinatorial technique was shown by the agreement of crystallographic and magnetic properties on two different sputtering setups. We observed an eightfold enhancement of the out-of-plane coercive field which was attributed to a shape anisotropy from column shaped grains observed in cross-sectional TEM. Angular dependence of switching fields agreed with the Kondorsky model for pinned 180° domain walls over a large vanadium composition range from 0.5 to 24 at% V with equal atomic concentrations of Fe and Co. This is the first time that quantitative evidence supports that Vicalloy is a pinning type permanent magnet. HiTp MOKE measurements were performed to accelerate the magnetic property screening of composition gradient and thickness gradient libraries. It was found that MOKE and VSM in-plane hysteresis loops had good agreement across large thickness and composition regions.

## Disclosure statement

No potential conflict of interest was reported by the authors.

## Funding

This work supported by European Commission [grant number NMP3-SL-2012-280670], Office of Energy Efficiency and Renewable Energy [grant number DE-AC02-07CH11358], Office of Science [grant number DE-AC02-76SF00515].
