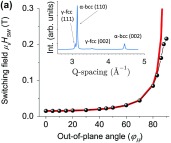# Erratum

**DOI:** 10.1080/14686996.2017.1314128

**Published:** 2017-04-10

Fackler SW, Alexandrakis V, König D, et al. Combinatorial study of Fe-Co-V hard magnetic thin films. Sci Technol Adv Mater. 2017;18:231—238.

http://dx.doi.org/10.1080/14686996.2017.1287520

When the above article was first published online, an incorrect version of Figure 5(a) was inadvertently included. The correct figure is shown below.

Taylor & Francis apologises for this error.